# Supplementary material for: Association between oral microbiome and seven types of cancers in East Asian population: a two-sample Mendelian randomization analysis
Source: Front Mol Biosci. 2023 Nov 21;10:1327893. doi: 10.3389/fmolb.2023.1327893 (PMC10702768; doi:10.3389/fmolb.2023.1327893)
Supplement: Supplementary file 1 [file DataSheet1.ZIP › Supplementary Materials/MR plots of saliva/saliva═╝/Lung Cancer/pheno.636_to_lung cancer_forest.pdf]

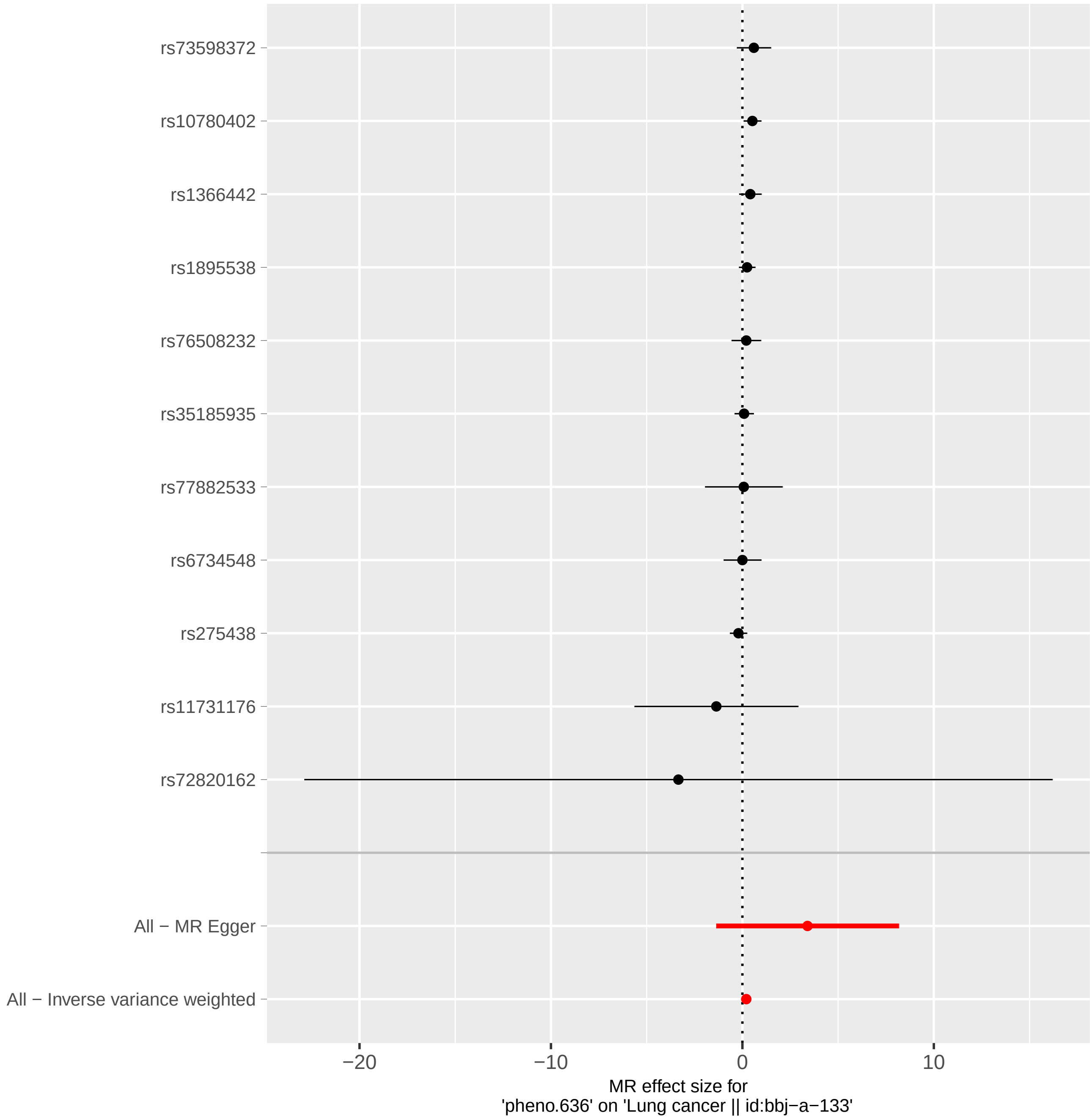

rs73598372
rs10780402
rs1366442
rs1895538
rs76508232
rs35185935
rs77882533
rs6734548
rs275438
rs11731176
rs72820162
All − MR Egger
All − Inverse variance weighted
−20
−10
0
10
MR effect size for
'pheno.636' on 'Lung cancer || id:bbj−a−133'
